# Association between Neutrophil Gelatinase-Associated Lipocalin and Fetal Hemoglobin Levels in Patients with Type 2 Diabetes Mellitus

**DOI:** 10.1155/2021/8383875

**Published:** 2021-10-20

**Authors:** Jong Weon Choi, Moon Hee Lee, Tatsuyoshi Fujii

**Affiliations:** ^1^Department of Laboratory Medicine, College of Medicine, Inha University, Incheon, Republic of Korea; ^2^Department of Internal Medicine, College of Medicine, Inha University, Incheon, Republic of Korea; ^3^Department of Internal Medicine, Tsukuba University Hospital Mito Clinical Education and Training Center, Mito Kyodo General Hospital, Ibaraki, Japan

## Abstract

The effect of neutrophil gelatinase-associated lipocalin (NGAL) on fetal hemoglobin (HbF) levels in diabetic patients is rarely investigated. This study is aimed at investigating the possible association between NGAL and HbF levels in type 2 diabetes mellitus (T2DM). A total of 160 patients with T2DM and 61 healthy individuals were evaluated. NGAL, HbF, tumor necrosis factor-*α* (TNF-*α*), interleukin-5 (IL-5), glycated hemoglobin (HbA1c), fasting plasma glucose (FPG), and urine albumin levels were measured. HbF levels were significantly higher in patients with elevated NGAL than in those without elevated NGAL (1.44% versus 0.94%, *P* = 0.001). High HbF was 2.3 times more prevalent in patients with elevated NGAL than in those without elevated NGAL. In addition, NGAL, TNF-*α*, and IL-5 levels were significantly higher in patients with high HbF than in those with low HbF; however, there was no significant difference in HbA1c and FPG levels between the two groups. HbF was positively correlated with NGAL (*r* = 0.275, *P* < 0.001), TNF-*α* (*r* = 0.256, *P* < 0.001), and IL-5 (*r* = 0.212, *P* < 0.001), but not with HbA1c and FPG. An elevated NGAL level led to a 1.27-fold increase in the prevalence of high HbF (odds ratio: 1.27, 95% CI: 1.03–2.51, and *P* < 0.001). The diagnostic efficacy of NGAL to identify an elevated HbF level was superior to that of HbA1c (area under the curve: 0.697, 95% CI: 0.609–0.786 versus 0.584, 95% CI: 0.488–0.681, and *P* = 0.022). In conclusion, enhanced NGAL production may be closely linked to elevated HbF in conjunction with proinflammatory cytokines in patients with T2DM.

## 1. Introduction

Fetal hemoglobin, also known as hemoglobin F (HbF), is a major hemoglobin fraction during the gestational period and in early infancy. HbF is produced by erythroid precursors from 12 weeks of pregnancy to the first 6 months of postnatal life [1]. HbF is almost entirely replaced by adult hemoglobin by the first year of life; thus, it constitutes less than 0.6% of the total hemoglobin of healthy adults [2]. However, HbF production can be reactivated under certain conditions in adulthood. For instance, pregnancy, starvation ketoacidosis, and some cancers lead to an increase in the production of HbF [3–5]. Additionally, HbF synthesis occurs under stress erythropoiesis conditions, such as hemolysis, hypoxia, and acute blood loss [6].

Augmented HbF production has also been reported in patients with diabetes [7]. However, the cause of HbF elevation is unclear. Different studies have reported varying results on HbF production in patients with type 2 diabetes mellitus (T2DM). In one study, the level of HbF was increased in patients with T2DM who had poor glycemic control, demonstrating a positive correlation with glycated hemoglobin (HbA1c) [8]. In contrast, in another study, there was no significant association between HbF and HbA1c in diabetic patients [9].

Neutrophil gelatinase-associated lipocalin (NGAL) is a small glycoprotein mainly secreted by activated neutrophils and expressed in various tissues, including the kidney, bone marrow, and adipose tissues [10]. NGAL has been regarded as an acute-phase protein because its blood level is increased under inflamed conditions [11]. Plasma NGAL is known to be elevated in diabetic patients due to renal dysfunction and inflammation [12]. Furthermore, NGAL plays a role in the development of anemia by inhibiting erythropoiesis in patients with systemic inflammation [13].

The clinical use of NGAL as a biomarker of acute kidney injury and as an indicator of the progression of renal disease has been widely investigated. However, there are limited studies on the role of NGAL in HbF production in diabetic patients. Therefore, this study investigated whether augmented NGAL production has any significant association with elevated HbF in patients with T2DM, particularly in conjunction with proinflammatory cytokines.

## 2. Materials and Methods

### 2.1. Subjects

This cross-sectional observational study was conducted in patients (*n* = 160) who were diagnosed with T2DM between January 2017 and December 2020 at Inha University Hospital. Their age ranged from 38 to 72 years (mean age: 59.8 years), with 89 patients of the male sex (55.6%). In the present study, only the subjects with newly diagnosed T2DM were included, who had no history of antihyperglycemic therapy or antihypertensive and lipid-lowering treatment. Age- and sex-matched healthy individuals (*n* = 61) without evidence of renal impairment, apparent inflammation, and medication were enrolled as the control group. Participants were randomly chosen to avoid selection bias. Patients with T2DM were diagnosed using the criteria of the American Diabetes Association [14]. The following subjects were excluded from the study because their conditions may affect HbF and NGAL levels: (a) those with a history of hemoglobinopathies; (b) those with renal dysfunction, acute blood loss, and present pregnancy; (c) those with sepsis or systemic inflammatory response syndrome; and (d) those receiving surgery or medication. Subjects with missing values in medical records and a fasting time < 8 h were also excluded from the analysis. Information on the status of cigarette smoking was obtained. The study protocol was approved by the institutional review board of Inha University Hospital (approval number: 2021-05-005). This study was conducted in accordance with the guidelines of the Helsinki Declaration.

### 2.2. Measurement of Laboratory Parameters

Blood samples were drawn into vacutainer tubes after overnight fasting. All samples were collected before treatment. Plasma, serum, and whole blood samples were used for analyses of NGAL, proinflammatory cytokines, and HbF and HbA1c, respectively. The fractions of HbF and HbA1c were measured by high-performance liquid chromatography using the G8 Glycohemoglobin Analyzer (Tosoh Bioscience, Tokyo, Japan). A high HbF level was defined as HbF > 1.0% [9]. Plasma NGAL was measured by fluorescence immunoassay using the Triage NGAL Test kit (Alere Inc., San Diego, CA, USA). The cutoff limit for an increase in NGAL levels was 150 ng/mL [15]. Proinflammatory cytokines were measured using enzyme-linked immunosorbent assay kits for tumor necrosis factor-*α* (TNF-*α*) (BD Inc., San Diego, CA, USA) and interleukin-5 (IL-5) (R&D Systems, Minneapolis, MN, USA) according to the manufacturer's instructions. High-sensitivity C-reactive protein (hsCRP) was measured using a chemical analyzer (Hitachi 7600; Hitachi, Tokyo, Japan). An increase in hsCRP levels was defined as >0.5 mg/dL based on the cutoff limit (95% confidence interval, CI) for hsCRP levels in healthy subjects. Hemoglobin and erythrocyte counts were obtained using an automated cell counter (ADVIA 120; Siemens, Forchheim, Germany). FPG, serum and urine creatinine (Cr), and urine albumin levels were analyzed using a chemical analyzer (Cobas 8000 C702; Roche, Mannheim, Germany). The albumin-to-creatinine ratio (ACR) was calculated using the following equation: ACR (*μ*g/mg Cr) = urine albumin (*μ*g/mL)/urine Cr (mg/dL). Microalbuminuria (MAU) and normoalbuminuria were defined as ACR = 30–300 *μ*g/mg Cr and ACR < 30 *μ*g/mg Cr, respectively [16]. The estimated glomerular filtration rate (eGFR) was estimated using the Modification of Diet in Renal Disease formula: eGFR = 186 × [serum Cr (mg/dL)]^−1.154^ × [age (years)]^−0.203^. An eGFR < 60 mL/min/1.73 m^2^ was regarded as renal dysfunction [17].

### 2.3. Categorization of Subjects

Subjects were categorized into two groups according to NGAL and HbF levels: patients with NGAL > 150 ng/mL (*n* = 76) and ≤150 ng/mL (*n* = 84) and patients with HbF > 1.0% (*n* = 41) and ≤1.0% (*n* = 119). Subjects were further stratified into several groups according to HbA1c and TNF-*α* levels: patients with HbA1c > 7.6% (*n* = 40) and <6.5% (*n* = 40) and patients with TNF − *α* > 95.2 pg/mL (*n* = 40) and <10.3 pg/mL (*n* = 40). These values were based on the upper and lower 25th percentiles of each parameter.

### 2.4. Statistical Analysis

Data were presented as mean ± standard deviation (SD) for normally distributed variables, median (interquartile range: IQR) for nonnormally distributed variables, and frequency (percentage) for categorical variables. The normality of data was confirmed by the Shapiro-Wilk test. Continuous variables with normal distribution were analyzed using Student's *t*-test. Nonnormally distributed data were analyzed using the Mann-Whitney *U*-test. The relationship between HbF and NGAL, glycemic parameters, and proinflammatory cytokines was assessed by multivariate linear regression analysis with adjustment for potential confounders, such as age, sex, body mass index (BMI), systolic blood pressure (SBP), eGFR, and smoking history. The relationship between elevated NGAL (upper 25th percentile) and the prevalence of high HbF was assessed by multivariate logistic regression analysis. A receiver operating characteristic (ROC) curve was analyzed to compare the diagnostic ability of NGAL and HbA1c to identify an elevated HbF level. Statistical analysis was performed with the SPSS software package (version 26; IBM SPSS Statistics, Armonk, NY, USA) and the MedCalc software package (version 20; MedCalc Software Ltd., Ostend, Belgium). Values of *P* < 0.05 were considered statistically significant.

## 3. Results

### 3.1. Clinical and Laboratory Characteristics of Patients

The concentrations of NGAL, HbF, and TNF-*α* were significantly higher in diabetic patients than in healthy individuals. Of the 160 patients, 76 (47.5%) patients had an elevated NGAL level, 41 (25.6%) patients had a high HbF level, 30 (18.7%) patients had MAU, and 89 (55.6%) patients had an elevated hsCRP level (Table 1).

### 3.2. HbF in Patients with and without Elevated NGAL

HbF levels were significantly higher in patients with elevated NGAL than in those without elevated NGAL. High HbF was 2.3 times more prevalent in patients with elevated NGAL than in those without elevated NGAL. In comparison with patients without elevated NGAL, patients with elevated NGAL had a significantly lower hemoglobin level. TNF-*α* and IL-5 levels were higher in patients with elevated NGAL than in those without elevated NGAL. However, there was no significant difference in the eGFR and prevalence of MAU between the two groups (Table 2).

### 3.3. NGAL, HbA1c, and Cytokines in Patients with Low and High HbF

Plasma NGAL level was 1.5 times higher in patients with high HbF than in those with low HbF. Proinflammatory cytokines and the prevalence of anemia were significantly higher in patients with high HbF than in those with low HbF. However, there was no significant difference in HbA1c, FPG, and kidney function between the groups (Table 3).

### 3.4. HbF Levels according to HbA1c and TNF-*α*

HbF levels were evaluated according to the quartiles of HbA1c and TNF-*α* levels. There was no significant difference in the HbF level between patients with HbA1c > 7.6% and those with HbA1c < 6.5%. However, HbF level was significantly higher in patients with TNF − *α* > 95.2 pg/mL than in those with TNF − *α* < 10.3 pg/mL (Table 4).

### 3.5. Association between NGAL and HbF Levels

The association between NGAL and HbF levels was assessed. When the subjects with the upper 25th percentile of NGAL (>180.5 ng/mL) were excluded from the elevated NGAL group, the HbF level of the elevated NGAL group was decreased to 1.07%, which was not significantly different from that of the nonelevated NGAL group. However, the hsCRP level was still higher in the elevated NGAL group than in the nonelevated NGAL group (Table 5).

### 3.6. Relationship between HbF and NGAL, Cytokines, and Glycemic Parameters

**Figure 1:**
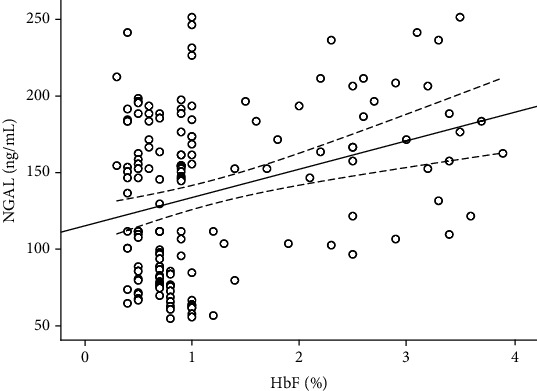
Scatter plots showing the relationship between HbF and NGAL levels in patients with T2DM. HbF levels are positively correlated with plasma NGAL concentrations (*y* = 18.52*x* + 113.6, *r*^2^ = 0.103, and *P* < 0.001).

After adjusting for potential confounders, HbF was significantly correlated with NGAL (*r* = 0.275, *P* < 0.001), TNF-*α* (*r* = 0.256, *P* < 0.001), and IL-5 (*r* = 0.212, *P* < 0.001). However, no significant correlation was observed between HbF and glycemic parameters, including HbA1c and FPG (Table 6). Scatter plots of the relationship between NGAL and HbF levels are shown in Figure 1.

### 3.7. NGAL and the Prevalence of High HbF

The relationship between elevated NGAL (upper 25th percentile) and the prevalence of high HbF was assessed by multivariate logistic regression analysis. An elevated NGAL level (>180.5 ng/mL) resulted in a 1.27-fold increase in the prevalence of high HbF (odds ratio: 1.27, 95% CI: 1.03–2.51, and *P* < 0.001) with adjustment for confounders (Table 7).

### 3.8. ROC Curve Analysis

**Figure 2:**
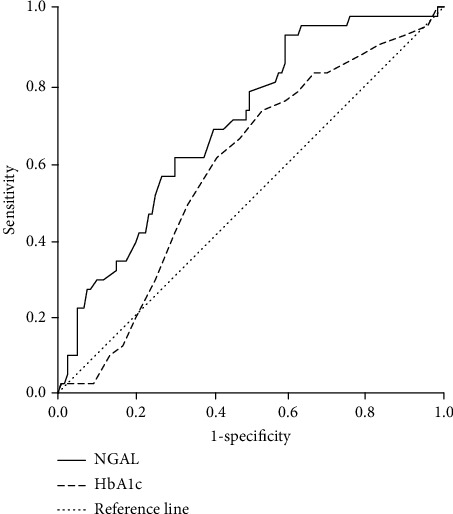
ROC curves showing the diagnostic abilities of NGAL and HbA1c to identify an elevated HbF level in patients with T2DM. Area under the curve (AUC) for NGAL is 0.697 (95% CI: 0.609–0.786), significantly larger than that for HbA1c (AUC: 0.584, 95% CI: 0.488–0.681, and *P* = 0.022).

The diagnostic values of NGAL and HbA1c to identify an elevated HbF level were investigated using a ROC curve analysis. The diagnostic accuracy of NGAL was significantly higher than that of HbA1c (area under the curve (AUC): 0.697, 95% CI: 0.609–0.786 versus AUC: 0.584, 95% CI: 0.488–0.681, and *P* = 0.022) (Figure 2).

## 4. Discussion

In the present study, the relationships between HbF, NGAL, HbA1c, and proinflammatory cytokines were assessed in patients with T2DM. The main findings of this study were that (1) high HbF was more prevalent in patients with elevated NGAL than in those without elevated NGAL, (2) HbF was more closely associated with NGAL, TNF-*α*, and IL-5 than HbA1c and FPG, and (3) NGAL elevation led to a 1.27-fold increase in the prevalence of high HbF. These findings suggest that elevated HbF may be due to enhanced NGAL production in conjunction with the release of proinflammatory cytokines during systemic inflammation in T2DM.

An increased HbF level can affect the measurement of HbA1c. HbF causes an overestimation of the level of HbA1c because HbF migrates closely with HbA1c in the analysis of hemoglobin using electrophoresis or ion-exchange assay [18]. On the other hand, an elevated HbF level can also falsely decrease HbA1c values in some immunoassay methods [19]. Therefore, it is necessary to check for the presence of HbF when monitoring diabetic patients. The prevalence of elevated HbF in diabetes ranges from 13% to 38% [7, 8]. In our study, 25.6% of patients with T2DM showed an increased HbF level. These contrasting findings may reflect differences in the patient population, presence of anemia, and severity of inflammation among studies. Additionally, there may be differences in the cutoff values for defining an elevated HbF level. For instance, various criteria have been used, such as >0.6% [8], ≥0.87% [4], >0.9% [20], and >1.0% [21]. In our study, HbF > 1.0% was chosen as the cutoff limit to specifically classify fetal-type erythropoiesis.

The cause of HbF elevation in patients with diabetes remains unclear. Nevertheless, several possibilities have been suggested. A study reported that a high HbF level was associated with insulin treatment, showing that HbF elevation was more often observed in insulin-treated patients than in noninsulin-treated patients and control subjects [22]. Pardini et al. [21] demonstrated that HbF was significantly elevated in diabetic patients with poor metabolic control, and it was positively correlated with HbA1c. However, in our study, no significant association was noted between HbF and HbA1c. In the present study, the level of HbF was assessed in patients who had no history of insulin therapy. Our results are consistent with those of a previous study showing that there was no significant difference in glycemic control and the duration of disease between diabetic patients with low and high HbF levels [23]. Based on these findings, insulin treatment or the status of glycemic control does not seem to sufficiently account for an elevated HbF level in patients with T2DM.

Hyperglycemia increases oxygen free radicals and shortens the life span of erythrocytes [24, 25]. NGAL inhibits the maturation of erythroid cells and induces the development of anemia [26]. Shrestha et al. [27] reported that NGAL was inversely correlated with erythrocyte counts, and that an increased NGAL level was associated with anemia. In another study, suppressed erythropoiesis was found to be associated with elevated HbF synthesis [28]. In our study, the level of HbF was higher in patients with elevated NGAL than in those without elevated NGAL. Similarly, NGAL was increased in patients with high HbF and was positively correlated with HbF. Furthermore, anemia was more prevalent in patients with high HbF than in those with low HbF. These results suggest that NGAL may play a crucial role in increased HbF production in diabetic patients in association with the occurrence of anemia.

To assess the effect of NGAL on HbF, subjects with high NGAL were excluded from the elevated NGAL group. Interestingly, after excluding subjects with the upper 25th percentile of NGAL, the HbF level of the elevated NGAL group was reduced to a level similar to that of the nonelevated NGAL group. Logistic regression analysis revealed that an elevated NGAL level was significantly associated with the prevalence of fetal-type erythropoiesis. Additionally, NGAL exhibited better diagnostic performance than HbA1c in identifying a raised HbF level. Therefore, it appears that an increased NGAL level, particularly at a moderately elevated level, may be closely linked to the upregulation of HbF. In our study, patients with preserved kidney function were enrolled to minimize the effect of renal function on both HbF and NGAL. There was no significant difference in the eGFR between the groups categorized according to NGAL and HbF levels. Therefore, it is unlikely that kidney function affected the results for HbF and NGAL.

Stress erythropoiesis is regarded as a stress response to anemia or hypoxemia [29]. In an experimental study, inflammation induced stress erythropoiesis by stimulating erythrocyte phagocytosis by splenic macrophages [30]. Proinflammatory cytokines inhibit erythropoiesis, which can lead to the development of anemia and induce stress erythropoiesis [31, 32]. T2DM is commonly accompanied by obesity, hyperlipidemia, and systemic inflammation. The prolonged exposure of endothelial cells to hyperglycemia causes vascular inflammation [33]. The present study evaluated the association between HbF levels and proinflammatory cytokines and glycemic parameters. There was no significant difference in the HbF level between patients with low HbA1c and those with high HbA1c. However, compared with patients with low TNF-*α*, patients with high TNF-*α* exhibited a significantly elevated HbF level. Additionally, TNF-*α* and IL-5 were significantly higher in patients with high HbF than in those with low HbF, demonstrating a positive correlation with HbF. These results suggest that enhanced HbF production in patients with T2DM may be more closely related to proinflammatory cytokines than elevated glycemic parameters. It is believed that the release of proinflammatory cytokines during systemic inflammation in T2DM promotes NGAL production, which generates stress erythropoiesis conditions by suppressing red cell maturation; thus, HbF synthesis may be initiated in patients with T2DM.

This study has several limitations. The status of hypoxemia in diabetic patients, which might affect HbF and NGAL levels, was not assessed. HbF levels were not measured in serial samples to evaluate possible disease progression. As our investigation was a cross-sectional study, the evidence for a cause-and-effect relationship between NGAL and HbF was limited. Small sample size, particularly in subgroup analyses, may limit sufficient statistical power. Despite these limitations, to the best of our knowledge, this is the first study to report the relationship between NGAL and HbF levels in patients with T2DM.

## 5. Conclusions

In summary, this study demonstrated that HbF levels were significantly higher in patients with elevated NGAL than in those without elevated NGAL. Furthermore, HbF was positively correlated with NGAL, TNF-*α*, and IL-5, but not with HbA1c and FPG. These results suggest that enhanced NGAL production may be closely linked to elevated HbF in conjunction with proinflammatory cytokines in patients with T2DM. Further studies are needed to validate our findings in larger randomized prospective trials.

## Figures and Tables

**Table 1:** Clinical and laboratory characteristics of diabetic patients and healthy individuals.

Parameters	Diabetic patients (*n* = 160)	Healthy individuals (*n* = 61)	*P* value
Clinical characteristics			
Age (years)	59.8 ± 7.5	60.3 ± 8.2	0.652
Sex (male, *n*, %)	89 (55.6)	32 (52.4)	0.670
BMI (kg/m^2^)	23.3 ± 3.7	21.6 ± 2.5	0.001
Smokers (*n*, %)	43 (26.8)	15 (24.6)	0.741
Duration of diabetes (years)	0.9 (0.3–1.2)	0 (0.0)	<0.001
Lipocalin			
NGAL (ng/mL)	145.0 (84.3–180.5)	63.5 (48.5–112.0)	<0.001
Elevated NGAL (*n*, %)	76 (47.5)	0 (0.0)	<0.001
HbF production			
HbF level (%)	1.18 ± 0.91	0.61 ± 0.32	<0.001
High HbF (*n*, %)	41 (25.6)	4 (6.5)	<0.001
Inflammatory parameters			
TNF-*α* (pg/mL)	40.6 (10.3–95.2)	15.2 (3.8–29.6)	<0.001
IL-5 (pg/mL)	14.1 (6.2–50.4)	3.9 (1.7–18.3)	<0.001
hsCRP (mg/dL)	0.72 (0.23–3.21)	0.10 (0.05–0.32)	<0.001
Elevated hsCRP (*n*, %)	89 (55.6)	0 (0.0)	<0.001
Glycemic parameters			
FPG (mmol/L)	9.8 ± 2.3	5.1 ± 0.2	<0.001
HbA1c (%)	7.16 ± 0.69	5.41 ± 0.28	<0.001
Kidney function			
eGFR (mL/min/1.73 m^2^)	81.5 ± 10.2	90.2 ± 6.5	<0.001
MAU (*n*, %)	30 (18.7)	0 (0.0)	<0.001

Data are expressed as mean ± SD, median (IQR), or frequency (%). BMI: body mass index; NGAL: neutrophil gelatinase-associated lipocalin; HbF: fetal hemoglobin; TNF-*α*: tumor necrosis factor-*α*; IL-5: interleukin-5; hsCRP: high-sensitivity C-reactive protein; FPG: fasting plasma glucose; HbA1c: glycated hemoglobin; eGFR: estimated glomerular filtration rate; MAU: microalbuminuria.

**Table 2:** HbF and proinflammatory cytokine levels in patients with and without elevated NGAL.

Parameters	Patients with diabetes		*P* value
	Elevated NGAL (*n* = 76)	Nonelevated NGAL (*n* = 84)	
HbF production			
HbF level (%)	1.44 ± 1.05	0.94 ± 0.68	0.001
High HbF (*n*, %)	28 (36.8)	13 (15.5)	0.002
Proinflammatory cytokines			
TNF-*α* (pg/mL)	53.1 (14.2–109.5)	30.6 (8.7–61.2)	<0.001
IL-5 (pg/mL)	21.8 (9.4–65.3)	10.3 (5.1–40.5)	<0.001
Anemia indices			
Hemoglobin (g/dL)	10.9 ± 2.4	13.1 ± 2.9	<0.001
Anemia (*n*, %)	34 (44.7)	15 (17.8)	<0.001
Kidney function			
eGFR (mL/min/1.73 m^2^)	80.4 ± 10.5	82.6 ± 11.2	0.203
MAU (*n*, %)	18 (23.7)	12 (14.3)	0.129

Data are expressed as mean ± SD, median (IQR), or frequency (%). HbF: fetal hemoglobin; TNF-*α*: tumor necrosis factor-*α*; IL-5: interleukin-5; eGFR: estimated glomerular filtration rate; MAU: microalbuminuria; NGAL: neutrophil gelatinase-associated lipocalin.

**Table 3:** NGAL, proinflammatory cytokines, and glycemic parameters in relation to the HbF level.

Parameters	Patients with diabetes		*P* value
	High HbF (*n* = 41)	Low HbF (*n* = 119)	
Lipocalin			
NGAL (ng/mL)	165.0 (120.5–195.0)	110.0 (78.0–165.0)	<0.001
Elevated NGAL (*n*, %)	28 (68.3)	48 (40.3)	0.002
Proinflammatory cytokines			
TNF-*α* (pg/mL)	70.2 (19.8–115.3)	31.8 (9.3–64.5)	<0.001
IL-5 (pg/mL)	24.5 (10.3–67.4)	11.2 (6.2–42.8)	<0.001
Glycemic parameters			
FPG (mmol/L)	10.1 ± 3.1	9.6 ± 2.7	0.327
HbA1c (%)	7.27 ± 0.61	7.12 ± 0.72	0.234
Anemia indices			
Hemoglobin (g/dL)	10.5 ± 2.6	13.2 ± 3.0	<0.001
Anemia (*n*, %)	21 (51.2)	28 (23.5)	<0.001
Kidney function			
eGFR (mL/min/1.73 m^2^)	80.6 ± 9.8	81.9 ± 10.4	0.485
MAU (*n*, %)	11 (26.8)	19 (16.0)	0.128

Data are expressed as mean ± SD, median (IQR), or frequency (%). NGAL: neutrophil gelatinase-associated lipocalin; TNF-*α*: tumor necrosis factor-*α*; IL-5: interleukin-5; FPG: fasting plasma glucose; HbA1c: glycated hemoglobin; eGFR: estimated glomerular filtration rate; MAU: microalbuminuria; HbF: fetal hemoglobin.

**Table 4:** HbF levels in patients with moderately elevated HbA1c and TNF-*α*.

Parameters	HbF level (%)	*P* value^∗^
HbA1c (%)		
Upper 25th percentile (>7.6)	1.12 ± 0.84	0.741
Lower 25th percentile (<6.5)	1.06 ± 0.78	
TNF-*α* (pg/mL)		
Upper 25th percentile (>95.2)	1.28 ± 0.97	0.011
Lower 25th percentile (<10.3)	0.82 ± 0.53	

Data are expressed as mean ± SD. ^∗^The upper 25th percentile values were compared with the lower 25th percentile values for each parameter. HbA1c: glycated hemoglobin; TNF-*α*: tumor necrosis factor-*α*; HbF: fetal hemoglobin.

**Table 5:** Association between NGAL elevation and HbF levels in patients with T2DM.

Parameters	Elevated NGAL (after excluding subjects with NGAL > 180.5 ng/mL; *n* = 36)	Nonelevated NGAL (*n* = 84)	*P* value
HbF (%)	1.07 ± 0.81	0.94 ± 0.68	0.367
HbA1c (%)	7.19 ± 0.62	7.08 ± 0.71	0.421
hsCRP (mg/dL)	0.71 (0.38–5.06)	0.36 (0.24–2.59)	<0.001
eGFR (mL/min/1.73 m^2^)	83.5 ± 10.1	82.6 ± 11.2	0.672

Data are expressed as mean ± SD or median (IQR). HbF: fetal hemoglobin; HbA1c: glycated hemoglobin; hsCRP: high-sensitivity C-reactive protein; eGFR: estimated glomerular filtration rate.

**Table 6:** Relationship between HbF and NGAL, glycemic parameters, and proinflammatory cytokines.

Variables	Correlation with HbF			
	Univariate		Multivariate^∗^	
	Standardized *β*	*P* value	Standardized *β*	*P* value
HbA1c	0.132	0.098	0.118	0.154
FPG	0.129	0.113	0.103	0.267
NGAL	0.322	<0.001	0.275	<0.001
TNF-*α*	0.305	<0.001	0.256	<0.001
IL-5	0.253	<0.001	0.212	<0.001
hsCRP	0.301	<0.001	0.245	<0.001

^∗^Adjusted for age, sex, BMI, SBP, eGFR, and smoking history. HbA1c: glycated hemoglobin; FPG: fasting plasma glucose; NGAL: neutrophil gelatinase-associated lipocalin; TNF-*α*: tumor necrosis factor-*α*; IL-5: interleukin-5; hsCRP: high-sensitivity C-reactive protein; HbF: fetal hemoglobin.

**Table 7:** Association between elevated NGAL and the prevalence of high HbF in patients with T2DM.

Elevated NGAL (>180.5 ng/mL)	Prevalence of high HbF	
	Odds ratio (95% CI)	*P* value
Unadjusted	2.53 (1.21–5.47)	<0.001
Adjusted for		
Age and sex	1.98 (1.16–4.03)	<0.001
Age, sex, and BMI	1.72 (1.12–3.64)	<0.001
Age, sex, BMI, and SBP	1.45 (1.09–3.07)	<0.001
Age, sex, BMI, SBP, eGFR, and smoking history	1.27 (1.03–2.51)	<0.001

NGAL: neutrophil gelatinase-associated lipocalin; BMI: body mass index; SBP: systolic blood pressure; eGFR: estimated glomerular filtration rate; HbF: fetal hemoglobin; CI: confidence interval.

## Data Availability

All data used to support the findings of this study are included in this article.
